# Patient Safety Policy in Long-Term Care: A Research Protocol to Assess Executive WalkRounds to Improve Management of Early Warning Signs for Patient Safety

**DOI:** 10.2196/resprot.3256

**Published:** 2014-07-15

**Authors:** Loes van Dusseldorp, Hub Hamers, Theo van Achterberg, Lisette Schoonhoven

**Affiliations:** ^1^Radboud University Medical CentreScientific Institute for Quality of HealthcareNijmegenNetherlands; ^2^HAN University of Applied SciencesFaculty of Health and Social StudiesNijmegenNetherlands; ^3^KU LeuvenCenter for Health Services and Nursing ResearchLeuvenBelgium; ^4^University of SouthamptonFaculty of Health SciencesSouthamptonUnited Kingdom

**Keywords:** governance, long-term care, executive board, patient safety, WalkRounds, soft signals

## Abstract

**Background:**

At many hospitals and long-term care organizations (such as nursing homes), executive board members have a responsibility to manage patient safety. Executive WalkRounds offer an opportunity for boards to build a trusting relationship with professionals and seem useful as a leadership tool to pick up on soft signals, which are indirect signals or early warnings that something is wrong. Because the majority of the research on WalkRounds has been performed in hospitals, it is unknown how board members of long-term care organizations develop their patient safety policy. Also, it is not clear if these board members use soft signals as a leadership tool and, if so, how this influences their patient safety policies.

**Objective:**

The objective of this study is to explore the added value and the feasibility of WalkRounds for patient safety management in long-term care. This study also aims to identify how executive board members of long-term care organizations manage patient safety and to describe the characteristics of boards.

**Methods:**

An explorative before-and-after study was conducted between April 2012 and February 2014 in 13 long-term care organizations in the Netherlands. After implementing the intervention in 6 organizations, data from 72 WalkRounds were gathered by observation and a reporting form. Before and after the intervention period, data collection included interviews, questionnaires, and studying reports of the executive boards. A mixed-method analysis is performed using descriptive statistics, *t* tests, and content analysis.

**Results:**

Results are expected to be ready in mid 2014.

**Conclusions:**

It is a challenge to keep track of ongoing development and implementation of patient safety management tools in long-term care. By performing this study in cooperation with the participating long-term care organizations, insight into the potential added value and the feasibility of this method will increase.

## Introduction

Good quality of care and patient safety require leadership involvement from both professionals and managers [1-6]. However, analyses of recent safety incidents in health care in the Netherlands show that there is a lack of governance on patient safety [7]. This is related to the fact that allocation of responsibilities for patient safety between professionals and managers is not clearly defined, and that board members, who have the final responsibility, lack leadership tools to improve and secure patient safety at an organizational level [7].

Currently available tools for the management of safety in health care are largely based on quantitative management information. Dashboards/scorecards and quality indicators emerge as a vital tool for hospital leaders who promote quality improvement within their organizations [5]. However, these tools do not paint the whole picture and, on their own, do not yield sufficient information to monitor quality and patient safety [8]. Vaughn et al [6] showed that having access to this information is only 1 of 5 characteristics of hospital boards that are associated with better quality index scores in hospitals. Spending more that 25% of time on quality issues, basing the senior executive’s compensation in part on quality improvement performance, identifying the chief executive officer as the person with the greatest impact on quality improvement, and engaging in a high level of interaction with the medical staff on quality strategy are the other 4 characteristics of hospital boards with better quality index scores.

In addition, Twijnstra and Gudde [9] identified the professional relationship between board members and professionals as an important precondition for safety policy in hospitals and in long-term care settings. This relationship should be based on mutual trust to allow the board to pick up on indirect signs (eg, conflicts between the medical staff or discontent of staff members). These so-called soft signals are important early warnings that something is wrong. They can supplement or confirm current management information and seem useful as a leadership tool for board members.

In 1999, the Institute for Healthcare Improvement initiated Executive WalkRounds. Frankel conceptualized these Executive WalkRounds as a tool to engage senior management in patient safety and to build a culture of safety within the organization [1,10]. Executive WalkRounds are conducted in patient care departments and provide an informal method for leaders to talk about safety issues in the organization with front-line staff and show their support for reporting errors [11]. According to Frankel [1,2,11], using these Executive WalkRounds allows senior executives of health care organizations to demonstrate commitment to building a culture of patient safety, provide opportunities to learn about patient safety, identify opportunities for improving safety, and establish lines of communication about patient safety with personnel. Research showed that Executive WalkRounds improved the safety culture in hospitals (eg, during 8 months, 39% of the patient safety issues were resolved) and added to the trust that the board was there to support and listen to professionals of all levels [2,12]. Executive WalkRounds are therefore considered to be an effective method to capture soft signals and a way to enhance the mutual trust between professionals and the board.

The majority of the research on patient safety and Executive WalkRounds has been performed in hospitals. It is therefore unknown how board members of long-term care organizations develop their patient safety policy. In addition, it is not clear if these board members use soft signals as a leadership tool and if so, how this influences their patient safety policy.

The aim of this study was to introduce and evaluate the method of Executive WalkRounds in long-term care organizations in the Netherlands to explore the added value and the feasibility of this method for picking up soft signals. In addition, this study aimed to identify how board members of long-term care organizations manage patient safety and to describe the characteristics of the boards.

## Methods

### Study Design and Setting

An explorative before-and-after study was conducted in long-term care organizations in the Netherlands between April 2012 and February 2014. We included 13 organizations that varied in size and were spread across rural and urban locations in the Netherlands (Table 1).

Before the introduction of Executive WalkRounds, data collection took place in the included long-term care organizations to identify the characteristics of boards of the organizations. Then, the intervention was implemented in 6 organizations during 1 year. After this period, the added value of managing soft signals on patient safety outcomes was investigated in all participating organizations.

**Table 1:** Characteristics of the participating organizations.

Health care sector	Size	Geographic location/urban or rural	No. of patients
Mental health	13 locations	West/urban	11,368
Mental health	3 regions	Mid East/urban	20,489
Mental health	6 locations	Mid South/rural	16,602
Mental health	20 locations	North/rural	18,029
Nursing home	19 locations	Mid West/urban	3389
Nursing home	3 locations	Mid/rural	246
Nursing home	2 locations	North West/rural	289
Nursing home	20 locations	Mid South/rural	2006
Nursing home	18 locations	Mid/urban	10,992
Physically and intellectually disabled	11 regions	Mid/urban and rural	2804
Physically and intellectually disabled	10 locations	Mid South/urban and rural	2523
Physically and intellectually disabled	43 locations	East/urban and rural	2047
Physically and intellectually disabled	400 locations	North/urban and rural	3188

### Ethical Aspects

The study was assessed by the Medical Ethics Committee of the district Arnhem – Nijmegen in the Netherlands. They concluded that, according to Dutch Law, this study was deemed exempt from their approval because it did not include collection of data at the level of patients.

### Participants and Sample

Convenience sampling was used to include a diverse group of long-term care organizations. We include 4 mental health care institutions, 5 nursing home and home care organizations, and 4 institutions for the physically and intellectually disabled. After written informed consent, the organizations were nonrandomly assigned to an intervention and a control group. The intervention group in which the method of Executive WalkRounds was introduced included 2 mental health care institutions, 2 nursing home and home care organizations, and 2 institutions for the physically and intellectually disabled. The other organizations (n=7) formed the control group and continued care as usual.

### Intervention

#### Overview

The development and introduction of the intervention had three stages: (1) modifying the original concept; (2) developing a standard script; and (3) introduction of the intervention.

#### Stage 1: Modifying the Original Concept

To promote the feasibility of the Executive WalkRounds in long-term care, we reviewed and modified the original concept developed by Frankel et al [2] (Table 2).

To promote the usability of the name and method we shortened the name to WalkRounds. We developed ground rules (Table 3) and translated the original “initial questions” for the WalkRounds developed by Frankel [2] and Cavanagh and Hulme [13] into Dutch. Furthermore, we added the possibility to extend the attendees (“with whom”) with patients, family, and relatives. Finally, the frequency was changed to monthly and WalkRounds were conducted by an interdisciplinary team at board level (eg, chairperson of the board, senior manager, senior quality improvement, and medical director).

**Table 2:** Original concept of WalkRounds.^a^

Who	Senior executives or vice presidents, the patient safety manager, a quality department director, the pharmacists assigned to the area, and a research assistant
Frequency /duration	Weekly/approximately 1 hour
Where	At the workplace of different areas of the hospital; eg, medical ward, surgical ward, emergency department or laboratory. In an open area to increase visibility.
With whom	Nurses and other available staff; eg, patient care assistants, and attending or resident physicians
Initial questions asked during the WalkRounds	Were you able to care for your patients this week as safely as possible? If not, why not? Can you describe how communication between caregivers either enhances or inhibits safe care on your unit? Can you describe the unit’s ability to work as a team? Have there been any “near misses” that almost caused patient harm but didn’t? Is there anything we could do to prevent future adverse events? What do you think this unit could do on a regular basis to improve safety? When you make an error, do you always report it? If you prevent/intercept an error, do you always report it? If you make or report an error, are you concerned about personal consequences? Do you know what happens to the information that you report? Have you developed any personal practices that you carry out to specifically prevent making errors? Have you discussed patient safety issues with your patients or their family? Do patients and families voice any safety concerns? What specific intervention from leadership would make the work you do safer for patients? What would make these executive WalkRounds more effective?
Recording	Comments on the questions are recorded on a worksheet.
Afterward	The senior executive briefly described a few of the important concepts that will lead to a safer environment. In addition, participants are asked to tell 2 other staff members about the WalkRounds.
Key factor	To help participants develop a sense of “psychological safety” allowing them to speak openly during the rounds, confidentiality and anonymity must be guaranteed.

^a^See Frankel et al [2].

**Table 3:** Ground rules of WalkRounds.

Organizations should decide whether to deviate from the principle of announcing the time and place of the WalkRounds.
An agreed WalkRound is not canceled by the WalkRound team. The ward/unit may cancel a WalkRound in case of exceptional circumstances, such as emergencies or incidents. In this case, a new WalkRound will take place within 1 week.
The maximum duration of a WalkRound is 60 minutes.
WalkRounds take place on the floor of a patient care unit; ie, office, recreation room.
All information discussed in WalkRounds is strictly confidential.

#### Stage 2: Developing a Standard Script

**Figure 1:**
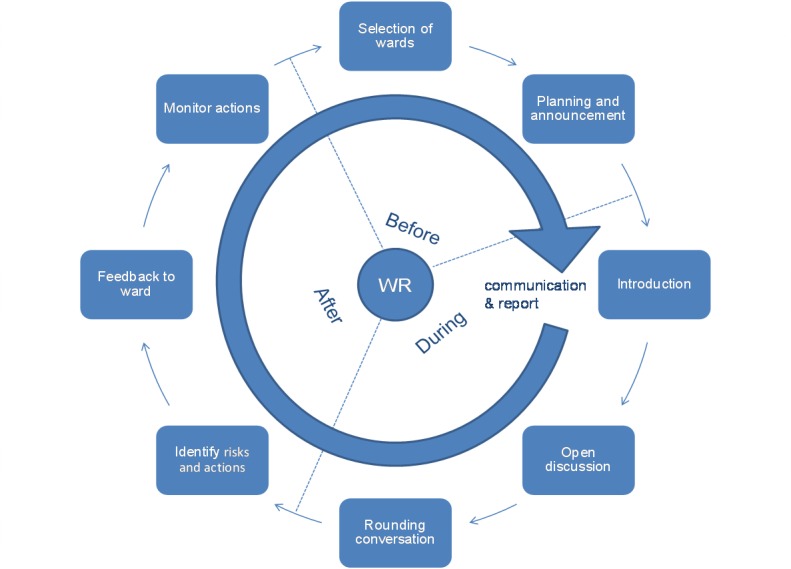
Phases during the WalkRound.

Based on the literature concerning Executive WalkRounds [2,3,11-14] we developed a standard script to facilitate the intervention. This standard scripts consisted of the introduction; the background; the procedure (ie, ground rules and the three phases; Figure 1); starting, final, and additional questions; and the reporting form.

We decided that each WalkRound lasted 30-60 minutes. An open discussion about patient safety was encouraged to hear the views of all present; staff, patients, family or relatives. From the original questions, we determined a standard starting question and a final question that we encouraged. These questions are “can you describe any near misses that almost caused patient harm, that occurred sometime during this week?”, and “what do you think this unit could do on a regular basis to improve safety? ”

After the WalkRound was completed, the WalkRound team reflected on the visit and reported the salient points such as the soft signals and safety risks, and agreed upon the improvement actions. Urgent problems had to be solved within 24-48 hours in collaboration with the board. The lead of the WalkRound team reported the patient safety issues to those responsible in the board on a regular basis.

Except for the ground rules, the starting and final questions, and the report form, the WalkRound teams had the opportunity to modify the standard script to their own setting and population.

#### Stage 3: Introduction of the Intervention

To introduce the intervention, each WalkRound team participated in a workshop of 3 hours in which they learned about the method and made working agreements to implement the WalkRounds in their organizations. To enhance the feasibility of the method, we did not enforce one standard script for the participating WalkRound teams. After the workshop, the WalkRound team decided which unit they would visit and ensured clear communication in the organization and the specific unit about aim and method. The WalkRounds were then announced, and during 1 year, the WalkRound team visited 6 different wards/units, including a follow-up visit per ward after 6 months.

#### Instruments

We use two types of instruments to collect data; instruments to collect data about the intervention (Table 4) and instruments to collect additional data (Table 5).

#### WalkRounds

To collect data on the WalkRounds (eg, duration, attendees, soft signals, and safety improvement activities) we developed a reporting form. To complement this form and collect data on the “psychological safety” (Table 1), a topic list was developed by the research team for collection of observational data about communication and ambience during the WalkRounds. We evaluated the feasibility of the method by conducting an open group interview with each WalkRound team. The opening question of each group interview was “How did you experience the WalkRounds?” Topics for this interview, advised by an expert in the field of improvement strategies, were feasibility of the method, facilitators and barriers, and results on patient safety, trust, and communication.

To identify how executive board members manage patient safety and to describe the characteristics of the boards, we translated and adjusted the original instrument “the Executive QI Survey,” of Joshi [15]. This survey consists of 34 questions about Board Engagement in Quality; 11 closed questions with response categories on a scale of 0-10, and 23 open questions. The translation and adjustment to the Dutch situation was performed by 2 members of the research team. Experts in the field of health care management, and quality and patient safety determined face validity of the adjusted questionnaire. Based on their feedback, the questionnaire was split in a written questionnaire and a semi structured interview. The questionnaire consisted of 22 questions: 13 closed questions with a response scale of 0-10, 2 closed questions that require a yes or no answer, and 7 open questions. For instance “On a 1-10 scale, how satisfied are you that the quality data the board reviews are the right measures for a comprehensive assessment of the organization’s real quality performance?” or “For a typical meeting, what are the major Board standing agenda items?” The interview lasted 60 minutes and was based on 21 open questions; for example, “How do patient perspectives get incorporated into the Board’s agenda for quality and safety improvement?”.

In addition, we collected information from the meetings of the executive boards, using a topic list. The researcher and the 2 supervisors of the project developed the topic list that was based on the characteristics of hospital leadership engagement in quality improvement [6] and control modalities of safety risks [16]. For information about the topics, see the description in Table 4.

To describe the variation in patient safety policy between the participating organizations, data about characteristics of the organizations are collected; that is, population, size, location, settings, number of clients and staff members, management vision, organizational structure, and allocation of responsibilities. To relate patient safety policy to better safety outcomes, we collected data from ZiZo, the Dutch framework of quality indicators [17]; especially the outcomes that focused on patient safety for 2011 and 2012. For systematic identification of new safety improvement activities, we used the framework developed by Hulscher et al [18]. This framework focuses on the content of the improvement activities.

**Table 4:** Data instruments for WalkRounds.

Instruments	Description
Reporting form	Information about duration, attendees, soft signals, risk assessment, and (the number of) safety improvement actions
Observation topic list	Information about communication and ambience
Open qualitative group interview	Feasibility of the method: experience in general, experienced results, barriers and facilitators, key factors regarding the influence of the WalkRounds

**Table 5:** Additional data instruments.

Topic	Description	Instruments
Characteristics of executive boards	Themes: amount of knowledge regarding quality and safety reports, agenda-setting, professionals, performance monitoring, responsibilities, values, and quality improvement activities	Questionnaire and semi structured interview (translated and adjusted from the Executive QI Survey [15])
Safety policy by executive boards	Frequency and duration of agenda items related to patient safety issues, signals about patient safety and instruments to collect these signals, the level of interaction with medical staff or health and safety committee on quality strategy, the safety culture, the allocation of responsibilities, and new safety improvement activities	Topic list (based on characteristics of hospital leadership engagement in quality improvement [6] and control modalities of safety risks [16])
Quality improvement activities	Information about the content of the activity, the participants, the executor, and whether the activity is based on soft signals detected during the WalkRounds	Framework of improvement activities [18]
Quality performance indicators	Indicators focusing on patient safety outcomes including the number and duration of seclusions and restraints, the duration of coerced medication, the percentage of patient falls, the percentage of medication incidents, the amount of weight loss, the percentage of patients with safety risks, and prevalence of safety incidents	Dutch framework of quality indicators [17] of mental health care, nursing home and home care, and care for the physically and intellectually disabled.

### Data Collection

#### Overview

Data collection took place at 3 time points: (1) at baseline; (2) during the intervention period; and (3) at follow-up after the intervention period.

#### Baseline

Baseline data concerning characteristics of boards and their safety policy were collected from April through June 2012. We interviewed the board members (n=23) individually, asked them to fill out the questionnaire, and studied the reports of the board meetings (n=13).

#### Intervention Period

The intervention period ran from July 2012 until June 2013. During this period, information about the WalkRounds was collected in 2 ways. First, the chair of each WalkRound team filled out the reporting form per WalkRounds. Second, every WalkRound was recorded on audiotape and observed by a member of the research team. In this way, we collected data about the communication and ambience. Because the 6 intervention organizations perform 12 WalkRounds each (1 per month), data from a total of 72 WalkRounds were gathered.

#### Follow-Up

Starting July 2013, data of the board were collected using the same instruments as used at baseline; for example, questionnaires, semi structured interviews, and by studying the reports focusing on patient safety and quality improvement, for July 2012 through June 2013. During this follow-up period, we also conducted open group interviews with the WalkRound teams to evaluate the method.

### Data Analysis

#### Overview

The analysis consists of two main parts: analysis of the WalkRounds, and analysis of the additional data.

#### Analysis of the WalkRounds

Quantitative data regarding the organization of the WalkRounds; for example, duration and attendees, are analyzed using descriptive statistics. The reported soft signals and improvement activities are analyzed using content analysis. The researchers create a coding framework based on codes generated by Montgomery [12] for the soft signals, and Hulscher [18] for the improvement activities. The coding framework contains the code and an operational definition. The reported data are coded using this framework. During the analysis, data are independently interpreted and coded by the researcher and research assistant. In case of disagreement, consensus is reached through discussion. The open group interviews regarding the evaluation of the WalkRounds are analyzed similarly. The coding framework for analyzing the group interviews is based on the items “added value and feasibility,” “trust and interaction,” and “implementation.”

#### Analysis of the Additional Data

Quantitative data regarding characteristics of the organizations, characteristics of the board, and the way board members manage patient safety are analyzed using descriptive statistics. For a comparison of the average number of safety improvement activities, within and between the intervention and control groups, *t* tests are computed; a *P*<.05 is considered statistically significant. In addition, *t* tests are computed to evaluate the influence of the intervention by comparing the patient safety performance indicators within and between the intervention and control groups.

Important texts that emerge from examination of the questionnaires and interviews are analyzed using qualitative content analysis. The reports are analyzed both quantitatively using descriptive statistics and qualitatively using open, axial, and selective coding [19], based on the coding manual. During each phase of coding, data are independently interpreted and coded by the researcher and research assistant. In case of disagreement, consensus is reached through discussion.

We use SPSS version 18 and ATLAS.Ti version 6.2 for the quantitative and qualitative data analysis, respectively.

## Discussion

### Challenges

The currently available tools for managing safety in health care do not appear to yield sufficient information to monitor patient safety [8]. In addition, soft signals seem useful as a leadership tool to supplement current management information [9]. Executive WalkRounds are thereby considered to be an effective method to capture these soft signals. However, to our knowledge, research on WalkRounds in which the board of long-term care organizations focus on soft signals has not yet been done. Therefore, the effect of WalkRounds on patient safety policy in long-term care is not known.

This study posed several challenges concerning studying the added value of managing soft signals by WalkRounds. First, because of the incentives of the national government to improve patient safety, organizations in the control group will also invest in patient safety during this study period. These organizations will probably invest in patient safety through other ways of quality improvement such as internal audits, or implementing a safety management system. Because of these initiatives, care as usual will change during the study period. In addition, organizations implementing WalkRounds may be required to use other safety promotion methods as well. Therefore, it will be difficult to compare the exact effects of WalkRounds.

Another challenge we want to discuss is the ongoing development of quality measuring instruments in the Netherlands. First, since 2013, nonprofit health care trade associations are responsible for developing their own quality indicators and data infrastructure because ZiZo, the Dutch framework of quality indicators, will no longer exist [20]. Possible consequences are that data on patient safety outcomes for 2012 are not yet available at the end of our study period, or data differ from 2011. Therefore, gathering data or comparing data of the influence of WalkRounds to safety outcomes might be difficult. Furthermore, development of vision on quality and patient safety results in a shift from quantitative management information toward process indicators as management tools. For example, the association of mental health care implemented a patient safety program between 2008 and 2011, which focused on process indicators to prevent or reduce adverse events. Implementation of the 7 goals (presence of protocols to prevent or reduce, eg, the number of restraints or seclusions, aggression, and suicide) of this program is still ongoing in the mental health care institutions. Since 2008, the association of nursing home and home care organizations also implemented patient safety improvement programs. They determined 5 focal points including implementing standards of responsible care, and improvement programs managing medication safety, preventing falls, or physical restraint. Furthermore, the association of the physically and mentally disabled defined 5 new issues for their patient safety agenda, including promoting the reporting of incidents, training risk awareness, and specific programs for example aimed at sexual harassment and abuse [21]. We consider this shift an opportunity for the long-term care to manage patient safety in a way that better fits the specific populations under care. On the other hand, because of these social dynamics in long-term care, it will be difficult to collect the same outcomes before and after the intervention period.

### Limitations

The methodological limitations of this study must be considered. First, there is a considerable diversity in the participating organizations; they vary in size, population, and rural and urban location. Although this may hinder comparisons in this study, we believe it also strengthens the evidence for the feasibility of the WalkRounds in the long-term care setting. Second, because of the small sample size of this study, the representativeness of the findings is at risk. Because exploration of the value and feasibility of WalkRounds is our primary goal, we believe that using a diverse sample will allow us to say something about all types of organizations, which in this case will contribute to representativeness. Third, due to the convenience sampling, potential confounding factors can threaten the internal validity [19]. The selection of the included organizations, and the nonrandom assignment to the intervention and control group can influence the outcomes of the study positively. Those who are willing to participate in this study may be atypical of the population due to their drive to improve patient safety policy. To minimize this threat, potential confounding factors and their impact on the interpretation of the study results will be identified by collecting baseline data of the organizations’ characteristics, the same data before and after the intervention period, and data of the trade specific quality measuring instruments.

Despite these limitations, we think that by performing this study in cooperation with the participating long-term care organizations, we will increase the insight into the potential added value of managing soft signals by WalkRounds and the feasibility of this method in long-term care.
